# Benefits of community-based education to the community in South African health science facilities

**DOI:** 10.4102/phcfm.v5i1.474

**Published:** 2013-04-23

**Authors:** Paula Diab, Penny Flack

**Affiliations:** 1 Department of Rural Health, College of Nursing & Public Health, Howard College Campus, University of KwaZulu-Natal, South Africa; 2 Discipline of Speech Language Pathology, College of Health Sciences, Westville Campus, University of KwaZulu-Natal, South Africa

## Abstract

**Background:**

Community-based education (CBE) is utilised by health science faculties worldwide to provide a relevant primary care experience for students and a service to underserved communities and, hopefully, to affect student career choices. The benefits to training institutions and students are well documented, but it may well be that communities, too, will be able to benefit from a more balanced partnership, where they are consulted in the planning of such training programmes.

**Method:**

An exploratory qualitative study was undertaken by three South African universities in the provinces of Limpopo, KwaZulu-Natal and the Western Cape. Focus group interviews were conducted in their local languages with groups of community leaders, patients and supervisors at community sites involved in CBE training. A thematic analysis of their views was undertaken with the aid of NVivo (version 9). Ethics approval was obtained from the respective universities and health care training sites.

**Results:**

Benefits to the community could be categorised into short-term and long-term benefits. Short-term benefits included improved service delivery, reduction in hospital referrals, home visits and community orientated primary health care, improved communication with patients and enhanced professionalism of the health care practitioner. Long-term benefits included improved teaching through a relationship with an academic institution and student familiarity with the health care system. Students also became involved in community upliftment projects, thereby acting as agents of change in these communities.

**Conclusion:**

Communities can certainly benefit from well-planned CBE programmes involving a training site - community site partnership.

## Abstrait

**Les avantages de l’éducation communautaire à la communauté dans les établissements sud-africains sciences de la santé**

**Présentation:**

Les facultés des sciences de la santé du monde entier ont recours à l’éducation communautaire afin de permettre aux étudiants de bénéficier d'une expérience en soins de santé primaire et de fournir un service aux communautés sous desservies et, espérons-le, afin d'influencer les choix professionnels des étudiants. Les bénéfices pour les institutions de formation et les étudiants sont bien documentés, mais il se pourrait bien que les communautés puissent également bénéficier d'un partenariat plus équilibré dans lequel elles seraient consultées dans la planification de ces programmes de formation.

**Méthode:**

Trois universités sud-africaines des provinces du Limpopo, du KwaZulu-Natal et du Cap occidental ont réalisé une étude qualitative préliminaire. Des groupes de discussion ont été organisés dans les langues locales avec des groupes composés de chefs communautaires, de patients et de superviseurs dans les sites communautaires participant à la formation à l’éducation communautaire. Une analyse thématique de leurs points de vue a été réalisée avec l'aide du logiciel NVivo (version 9). Les comités d’éthique des universités et sites de formation aux soins de santé respectifs ont approuvé l’étude.

**Résultats:**

Les bénéfices pour la communauté peuvent être classés en bénéfices à court terme et bénéfices à long terme. Les bénéfices à court terme relevés sont les suivants: une meilleure prestation de services, la réduction des transferts en hôpital, les visites à domicile et les soins de santé orientés vers la communauté, une meilleure communication avec les patients, et un plus grand professionnalisme de la part des professionnels de la santé. Les bénéfices à long terme sont quant à eux un meilleur enseignement grâce à l’établissement d'une relation avec une institution universitaire et la familiarité des étudiants avec le système de santé. Les étudiants participaient également à des projets de développement communautaire, tenant ainsi lieu d'agents du changement au sein de ces communautés.

**Conclusion:**

Les communautés peuvent certainement bénéficier de programmes d’éducation communautaire bien planifiés impliquant un partenariat entre un site de formation et un site communautaire.

## Introduction

Community-based education (CBE) is described by Bean1 as a ‘win-win’ programme as it provides both the training institution and the service site with additional resources. In CBE, service is typically provided to the community by students who are placed in service learning sites. In the case of health science students these can include community health centres, schools, non-governmental organisations (NGOs), primary health care clinics and rural hospitals.2 Service is provided to the underserved in the community, and students get experiential and contextual learning that is relevant to their education, and that has the potential to influence their ultimate career discipline choice.^1, 3^

The benefits to the students involved with CBE are well documented in the literature and include improved practical knowledge and skills and a more positive attitude towards their patients and colleagues.^1, 4, 5, 6, 7, 8, 9, 10^ The primary benefit is that the students’ exposure to the real-world context allows them to understand the relevance of their training.^1, 2, 4^ Students learn about health systems and the inequities in and barriers to health care that are experienced by many people.6 Because of their exposure to different patient populations students also develop improved cultural awareness and cultural competence.^5, 6, 7, 8^

Although the benefits of CBE to students are well documented, very little evidence of the benefits of CBE to communities has been documented.

### Background

Hunt et al. identify a gap in the literature with regard to the reciprocal nature of the partnership in CBE.^6^ There is little focus on the collaborative nature of this partnership and, indeed, on whether it can be viewed as such. What is described in the literature is the contribution of students, and the benefits they derive from CBE. This raises an important concern about the nature of the partnership between the training institution, the service site and the community. Kristina, Majoor and Van Der Vleuten suggest that there is a greater benefit for students than for communities,^11^ but Williams, Reid, Myeni suggest that although communities do not benefit from CBE in the same way as students do, there is potential for a more balanced partnership where the community is consulted in planning a health programme that is relevant to that community's particular needs.^12^

Benefits to the community include both immediate and long term benefits. These range from increased *access to service* as a result of student placement in the typically overburdened community sites,^8^ *improved patient care* because of the quality of service offered as a result of updated practice techniques^1, 5, 8^ and clinic staff being able to *interact with the university*.^4, 5^

Students developing an increased *interest in rural practice* after graduation because of a positive undergraduate experience in rural communities constitute a long-term benefit to the community, as they often return to the community for their final year of community service.^3, 4, 5, 13^ A further benefit for communities is that by exposing students to the realities that communities face with regard to the lack of access to care and the social determinants of disease within communities, these students are more likely to become *advocates for change*.^5, 14, 15^ The service delivery model adopted by CBE programmes also benefits communities in that it supplies care that includes home visits and community orientated primary care.

#### Significance of work

This study is part of a larger study on community-based education (CBE). Instead of focusing on the benefits to students, this paper sheds light on how a community can benefit from CBE. In this paper the term community is used for the people living in the community and receiving the service provided by the health science students, as well as for the students’ trainers and/or supervisors who are based at the community service sites.

## Ethical considerations

Ethics approval was obtained from the Medunsa Research and Ethics Committee of the University of Limpopo – Medunsa Campus (Ethics Clearance Number: MREC/M/20/2010:IR), the Humanities and Social Sciences Research Ethics Committee of the University of KwaZulu-Natal (Protocol Reference Number: HSS/0210/010) and the Research Ethics Committee of the University of the Western Cape (Registration Number: 10/7/15). Permission to conduct the study was obtained from the senior management of the various study sites in each province. Ethical principles of autonomy, beneficence, non-maleficence and justice, as stipulated in the ethical guidelines of the Medical Research Council, were upheld throughout the study.

## Research method and design

### Design

This study forms part of a larger, qualitative, exploratory study of views and attitudes towards, and recommendations for CBE that was conducted across three South African universities: the University of Limpopo, the University of KwaZulu-Natal and the University of the Western Cape, by members of the CHEER research collaboration. (CHEER is the Collaboration for Health Equity through Education and Research, a group of Health Science Faculty academics from nine South African universities.)

### Materials

Focus group interviews comprising an average of five participants were conducted in the health care facilities used as training sites by the respective universities. Participants included community leaders of participating health care facilities, health care providers involved in student supervision, and community members (patients) who were the recipients of the care rendered by the students. An interview schedule was developed collaboratively and it was then used as a guide for the focus group interviews.

### Analysis

The interviews were audio taped and transcribed verbatim, translated into English by independent research assistants at each university who were knowledgeable in the local languages (Setswana, Sepedi, isiZulu, isiXhosa and Afrikaans) and checked for accuracy. Data analysis was aided by the use of NVivo (version 9).

## Discussion of results

### Immediate benefits to the community

Two of the main effects of the students’ presence, as perceived by an overwhelming majority of the participants in the interviews, were related to service delivery: they provided assistance by providing extra hands and alleviating the day's patient load. Although the students were not fully qualified, they could, depending on their level of training, consult with patients under supervision from a senior colleague or carry out basic, time-consuming tasks. These benefits were reported universally by all interviewees, whether community members, patients or staff:

‘… help with the shortage of doctors’. (Community leader, KZN)

‘I think we need student doctors because we hear from the radio that there is a shortage of staff’. (Community leader, Limpopo)

‘… immediately as you arrive at the OPD, the [*student*] doctors attend to you’. (Patient, KZN)

‘It is necessary that they come in places like these because there are so many sick people that need assistance’. (Patient, KZN)

‘Queues are better’. (Supervisor, Limpopo)

‘… if it wasn't for them, it will mean that these patients would have waited the whole night’. (Supervisor, Limpopo)

Because the students examined patients at the site, referral of patients to hospitals was reduced. This, in turn, would also have decreased the congestion at the hospitals:

‘Now patients who are seen by the doctor here do not [*need*] to go to the hospital’. (Supervisor, Limpopo)

‘… we are happy that our pensioners and chronics [*sic*] will not be going to the hospital but will be seen here’. (Supervisor, Limpopo)

‘The coming of doctors to the clinic has made a lot of change. They have decreased the long queues in the hospital, there used to be so much congestion …’. (Supervisor, Limpopo)

The benefit of home visits was also seen as important by the communities as:

‘[*The students*] gain knowledge about the life of people on the farms’. (Supervisor, Limpopo)

It was deemed important, too, for students to:

‘see the environment, the socio-economic and geographical needs of that community and that family’. (Supervisor, Limpopo)

Both these aspects are important learning outcomes for students and the communities themselves benefit greatly from a health-care provider who understands their environment and context. Knowledge of the community in which they work was highlighted by many as playing a vital role in delivering a good service to that community:

‘… they receive first-hand information [*about*] the socio-economic problems of the people. They learn a lot from the clinic, things that they don't learn from the best hospitals’. (Supervisor, Limpopo)

Community-orientated primary care (COPC), which is another key learning outcome, resulted in specific benefits to the community in that students would:

‘actually go to [*the patients’*] homes’. (Supervisor, KZN)

‘do community-based studies’. (Supervisor, Limpopo)

and then ‘follow the patient for a certain period at home’. (Supervisor, Limpopo)

Community leaders thought that a generic experience of how communities function was just as important as practical clinical training constitutes both an immediate benefit and a long-term benefit for the community. The community benefits immediately when students begin to apply what they have learnt about how communities function to their clinical practice. There is also the long-term benefit when students return to the same communities as graduates and bring with them their prior experience of the community structure and functioning:

‘You need to change and adapt to the circumstances, you need to prioritise the situation because when you are a doctor you are going to be in charge … you make a judgement call’. (Community leader, KZN)

Within the context of the health care institution, many of the patients commented that they:

‘don't see a difference’ between permanent staff and the students, ‘because they [*the students*] treat us well’. (Patient, Limpopo)

Sometimes students were seen to offer a better service:

‘… they [*patients*] seem to get better treatment and a quicker [*service*]’. (Supervisor, Limpopo)

Generally students were seen in a very positive light by community leaders and patients alike:

‘… they checked me more than the sisters do …’. (Patient, Limpopo)

‘…they did not rush anyone, they took a long time checking me …’. (Patient, Limpopo)

‘They are patient with us … they want to know your problems so that they know the cause of your illness’. (Patient, Limpopo)

‘The students take time explaining our problems and we are really satisfied’. (Patient, Limpopo)

A community leader realises that:

‘… in life as a person you need to be simple, down to earth that is what doctoring is all about - empathy and feelings’. (Community leader, KZN)

And that the students are able to provide such care. Patients often preferred to see a student as they:

‘… can freely talk to [*them*]’. (Supervisor, KZN)

And because ‘… the student doctors give them more attention … the students go for holistically managing the patient so it makes the patient feel more important and they go home satisfied …’. (Supervisor, Limpopo)

In addition to community members commenting positively on the nature of the care provided by students, they also expressed satisfaction with the quality of the care in terms of the skills demonstrated by the students. Because students are not under the same time constraints as their professional colleagues, they are able to concentrate more on skills such as communication and professional behaviour. A clinician who is sensitive to language and cultural differences is indeed highly valued:

‘I was praying to get someone who speaks Zulu and was lucky so that we could understand each other’. (Patient, Limpopo)

Some patients preferred a:

‘nurse to be in the cubicle [*to*] interpret … but it does happen for the doctor to do everything with the patient alone …’. (Community leader, KZN)

A vital adjuvant to understanding language and breaking down barriers in communication is:

‘…observ[*ing*] the body language…’. (Community leader, WC)

‘Another part is the different cultures. One of my staff was Zulu speaking and when they enter they will semi-bow, they will not look me in the face, it is not out of fear but it is their culture - someone who is senior to them or older to them, they do not look them in the face’. (Community leader, KZN)

### Long-term benefits to community

One of the key benefits to the community site is the staff's interaction with an academic institution on a long-term basis. The university is able to provide up-to-date teaching and to provide in-service education, not only on clinical topics but also in supervisory teaching and support to the facilitators at the sites:

‘I see the university as an academic institution with lots of resources in terms of new guidelines, new thinking. Generally the university has access to journals, for instance. I do not know if that can be possible to extend that to the hospitals that supervise the students which will benefit us as doctors working here … also to come and do continuous development talks here for us – that will be great, we will feel we get something back from the university and I think doctors will be very much more keen to interact with the students and to tutor them’. (Supervisor, KZN)

‘I think with the knowledge they have attained from the university they bring it back to the patients and they help the patient’. (Supervisor, KZN)

It is important for students to become familiar with the health care system; this holds long-term benefits for the communities in which they will eventually work. By introducing students to community-based care at an early stage in their careers, they become more accustomed to the system and are better able to operate within it:

‘The balance we should look at is have we got the capacity to support and supervise students for the spin-off that by the time they come as final years, if they spend a month here, that is going to be a very productive month if we have got the same students?’. (Supervisor, KZN)

It was felt that if students could gain knowledge of the community in which they found themselves, return to service would be a feasible option for them:

‘… tomorrow when this student [*has*] qualified, they know exactly because they were exposed’. (Supervisor, Limpopo)

‘… even after they have completed their studies, they usually work here. It's nice to work with them, they come back and work here’. (Supervisor, Limpopo)

‘… for the students it must be a valuable learning experience and the benefits for the community, I see in trying to get young doctors to return to this area’. (Supervisor, KZN)

‘… they come back during their community service year to actually go back to the same hospital they were during [*their studies*]’. (Supervisor, KZN)

Communities also felt that students should not only do:

‘doctor type of issues’. (Community leader, KZN)

As interacting with patients, observing how they were treated in the hospital and observing management and administrative issues was also an important part of their learning experience. Students could then:

‘come up with inventions that will assist us to better manage our patients …’. (Community leader, KZN)

The community benefits even more when students engage with the community in community upliftment projects and health promotion campaigns:

‘… [*teaching*] the people to knit. They taught the mothers so that they could generate an income and they taught them how to plant and till a small piece of land and see how wonderful it was’. (Community leader, WC)

‘We also make them engage in community-based activities, like last year they participated in the women's day, they go for pap smear taking, HIV counselling and testing and they also did massage’. (Supervisor, WC)

Lastly, a community benefits when students act as advocates for that community:

‘I would be happy if when students come here [*they*] submit bursary forms so our children can apply. Here is rural and we can't go to Medunsa and we also do not have access to computers’. (Community leader, Limpopo)

‘We really need the awareness. They [*Dept of Health*] place adverts outside here that we want physio, OT but it is not easy to access that so awareness can work’. (Community leader, KZN)

‘… [*they*] had to go out to schools and talk to children … it had a positive impact on the society’. (Supervisor, KZN)

#### Sustainability

Communities were pleased to be part of this important task of training young professionals:

‘We feel happy for our children as we educate them. We want them to continue until they get their degrees. We are happy for it’. (Patient, KZN)

They also saw the need to ‘make it easy for [*the students*] … to retain the staff … so if you have good, talented students, they will grow and if they grow in their own country, they will help their own people and as a government if we can achieve that obviously the health system is going to improve’. (Community leader, KZN)

‘The programme should continue because even the qualified ones, the time will come for them to retire so it is good when the youth study’. (Supervisor, KZN)

There is evidence that there are both immediate and long-term benefits for the community when CBE is implemented. However, the long-term success or failure of a CBE programme rests on the nature of the agreement between the stakeholders.^7^ One of the important ways in which to ensure sustainability is to involve the community from the start of the planning process. An ideal situation would be for health care workers to partner with the communities where student placement occurs.^16^ In our study the concern of some community leaders indicated the need for a dialogic relationship between the two partners:

‘… so there has to be a discussion between the two parties and we must settle on a workable solution … it can be a new arrangement … to consider’. (Community leader, WC)

## Limitations of the study

This study did not look at the views and attitudes of students or consider their experiences of CBE. Further research is recommended to document student experiences in the community and to ascertain how programmes can be designed to meet their needs as well as those of the community.

## Recommendations

Community leaders are important stake-holders in the education of students within communities. In this study, they reported that they had not been informed of the student training programme at the training sites. This omission made them feel undermined. They were therefore not in a position to inform the communities they represented. The negative effect of the lack of communication with community leaders and therefore with the community at large is the missed opportunity of shared ownership of the programme by both community and health care officials.

Joint ownership with communities has been found to play a significant role in student learning in ‘the community as a classroom’.^17^ The relationship between communities and centres of higher learning tends to be one-sided in that communities are mostly the passive recipients of what the students offer.^18^ By forming a partnership with communities, we can ensure that more *relevant health care* is delivered to the community and that the focus shifts towards more *community-based health care*. By creating a positive, productive and effective service to the community, we can also foster a heightened *interest in rural practice* and encourage students to identify community needs and become *agents of change* within those communities providing further, related benefit to the communities.

In order to benefit all parties concerned, it is recommended that a formal agreement be instituted to protect the concerns of both parties as well as to ensure that the scope of the agreement is fulfilled by both parties. Balancing service delivery to the community and teaching demands must also be considered in such an agreement.

The onus is on the university to assist with supporting and training the supervisors.

## Conclusion

The benefits of CBE to students are well documented; the benefits to the communities themselves are often overlooked, however. A qualitative investigation such as this study provides insight into the views and experiences of communities in various districts in South Africa. This study aimed to identify the benefits of CBE to the communities involved in order to promote a more equal partnership with training institutions.

As presented above, these include the immediate benefits of improved service delivery, increased access to service and improved patient care, as well as the long-term benefits of encouraging health professionals to community practice and to return to service in that area. In order to ensure sustainability and maximum benefit for all concerned, the communities should be involved in the initial planning of such activities. A formal needs analysis should take place and learning activities aligned to the needs of the community, as identified by the community and the academic facilitators alike. A formal plan should support these activities and clearly define the expectations on both sides and the mutual benefits expected. When the benefits to communities are documented and when there is co-operation between communities and training institutions, CBE can become more marketable to other communities and the scope for such learning enhanced. Training institutions are under enormous pressure to provide enhanced teaching platforms for their students and certainly CBE, if presented in an organised and mutually beneficial manner, can provide some of the solutions.
